# Pseudo almost periodic solutions for shunting inhibitory cellular neural networks with continuously distributed delays

**DOI:** 10.1186/s13660-017-1515-8

**Published:** 2017-10-02

**Authors:** Yueming Lu, Desheng Ji

**Affiliations:** 1 Department of Applied Mathematics, Harbin University of Science and Technology, Xue Fu Road, Harbin, 150080 P.R. China; 2 Department of Mathematics and Applied Mathematics, Heilongjiang University, Xue Fu Road, Harbin, 150080 P.R. China

**Keywords:** 46N10, 46N99, shunting inhibitory cellular neural networks, pseudo almost periodic solution, asymptotical stability, Halanay inequality

## Abstract

The shunting inhibitory cellular neural networks with continuously distributed delays and pseudo almost periodic coefficients are considered. First, we make a generalization of the Halanay inequality, and then establish some sufficient conditions for the existence and asymptotical stability of pseudo almost periodic solutions. Finally, a numerical simulation is presented to illustrate the theoretical results.

## Introduction

The shunting inhibitory cellular neural networks (SICNNs) was introduced by Bouzerdout and Pinter [1] in 1993. They presented the biophysical interpretation of the networks along with stability analysis of their dynamics. In the last two decades, SICNNs have been extensively applied in psychophysics, perception, robotics, adaptive pattern recognition, vision etc. In the feasibility analysis of these applications, the stability of networks is of the greatest importance. Recently, many sufficient conditions have been obtained to ensure the global exponential stability, locally asymptotical stability of neural networks via the LaSalle invariance principle, Lyapunov functionals and differential inequality techniques (see [2–5] and the references therein).

Besides stability, there are two other important factors: delays and variance of coefficients, have to be considered in real applications. Due to the finite speed of information processing, the time delays often exist in various neural networks. The time delay usually changes the dynamical properties of neural networks, for example, the time delay often cause oscillation, divergence or instability of neural networks. Thus it is important for us to consider the delay effect on the stability of delayed neural networks. For example, in order to deal with moving images, one must introduce the time delays in the signal transmission among the cells. Recently, many authors have studied various neural networks with distributed delays [6–9], which it is more appropriate to incorporate under the circumstances that neural networks have a spatial extent due to the presence of a multitude of parallel pathways with a variety of axon sizes and lengths and the signal propagation is instantaneous and cannot be modeled with discrete delays [10].

It is more realistic to consider non-autonomous neural networks in the real world, because many disruptive factors, such as a voltage fluctuation, may vary coefficients (decay rate and connection weight) and input signals of neural networks. Such functions, which are both appropriate to model the practical situation and available with some mature mathematical theories, are of great interest as regards the adoption as coefficients and input signals of neural networks. In the last decade, many authors studied the almost periodic solutions of kinds of neural networks including SICNNs (*e.g.*, see [2, 11–16] and the references therein). It is worthwhile to mention that pseudo almost periodic neural networks attracted much attention [9, 17–19] very recently. The pseudo almost periodic functions introduced by Zhang in [20–22] are a natural generalization of almost periodic functions and have been widely applied in many areas. We refer the reader to [23–27] for some recent work on this.

In view of the above-mentioned facts, we consider the SICNNs with continuously distributed delays as follows:

1 $$\begin{aligned} \frac{\mathrm{d}x_{ij}(t)}{\mathrm{d}t}=-a_{ij}(t)x_{ij}(t)-\sum _{C_{kl}\in N_{r}(i,j)}C_{ij}^{kl}(t) \int_{0}^{+\infty }k_{ij}(u)f \bigl(x _{kl}(t-u) \bigr)\,\mathrm{d}u\, x_{ij}(t)+L_{ij}(t), \end{aligned}$$

where $C_{ij}$ denotes the cell at the $(i,j)$ position of the lattice, $N_{r}(i,j)$ denotes $C_{ij}$’s *r*-neighborhood $\{C_{kl}:\max (\vert k-i\vert ,\vert l-j\vert ) \leq r,1\leq k \leq m, 1\leq l \leq n \}$, $x_{ij}(t)$ is the activity of the cell $C_{ij}$, $L_{ij}(t)$ is the external input to $C_{ij}$, $a _{ij}(t)>0$ represents the passive decay rate of the cell activity, $C_{ij}^{kl}(t)$ is the connection or coupling strength of postsynaptic activity of the cell transmitted to the cell $C_{ij}$, and the activity function $f(x)$ is a continuous function representing the output or firing rate of the cell, $k_{ij}(u)$ is the kernel function determining the distributed delays at the cell $C_{ij}$, $i \in \{1,2,\ldots,m\}$, $j \in \{1,2,\ldots,n\}$. The decay rate $a_{ij}(\cdot)$, coupling strength $C_{ij}^{kl}(\cdot)$ and external input $L_{ij}(\cdot)$ are all assumed to be pseudo almost periodic (pap, for short).

The initial conditions associated with system (1) are of the form $x_{ij}(s)=\varphi_{ij}(s)$, $s\in (-\infty,0]$, where $\varphi =\{\varphi_{ij}(t)\}$ is bounded and continuous on $(-\infty,0]$. We make the following assumptions:

($T_{1}$)
$a_{ij}(\cdot)\in \mathit{PAP}(\mathbb{R};\mathbb{R})$ is ergodic, and $0<\underline{a}_{ij}=\inf_{t\in \mathbb{R}}a_{ij}(t)$.
($T_{2}$)
$L_{ij}(\cdot)$, $C_{ij}^{kl}(\cdot)\in \mathit{PAP}(\mathbb{R}; \mathbb{R})$ and $L^{+}_{ij}=\sup_{t\in \mathbb{R}}\vert L_{ij}(t)\vert $, $0\leq C_{ij}^{kl}(t)\leq \bar{C}_{ij}^{kl}$.
($T_{3}$)
*f* is Lipschitz continuous, $\vert f(u)-f(v)\vert \leq \mu \vert u-v\vert $, $\forall u,v \in \mathbb{R}$ for a constant *μ*.
($T_{4}$)
The delay kernel $k_{ij}$ belongs to $L^{1}(\mathbb{R}^{+})$ and $\int_{0}^{+\infty }\vert k_{ij}(s)\vert \,\mathrm{d}s\leq \hat{k}_{ij}$ for some positive constant $\hat{k}_{ij}$.
($T_{5}$)
$0<\delta,q<1$, where $M_{f}=\Vert f\Vert = \sup_{t\in \mathbb{R}}\vert f(t)\vert $, $L=\max_{(i,j)} \{\frac{L ^{+}_{ij}}{\underline{a}_{ij}} \}$, and
  $$\delta =\max_{(i,j)} \biggl\{ \frac{M_{f}\hat{k}_{ij}\sum_{C_{kl}\in N_{r}(i,j)} \bar{C}_{ij}^{kl}}{\underline{a}_{ij}} \biggr\} ,\qquad q= \delta \biggl( 1+\frac{\mu L}{(1-\delta)M_{f}} \biggr). $$

Under these assumptions, we have two innovation points which are supplements of the existing literature. One is that we assume the decay rate $a_{ij}(\cdot)$ is pap, while it was all assumed to be almost periodic in the literature listed above. The solution of (1) is not necessarily pap for a general pap function $a_{ij}(\cdot)$. To compensate for this, we assume $a_{ij}(\cdot)$ also is ergodic [20]. The other one is that we remove the assumption that there exists a positive number $\lambda_{0} > 0$, such that

$$\int_{0}^{+\infty } \bigl\vert k_{ij}(s) \bigr\vert \mathrm{e}^{\lambda_{0} s}\,\mathrm{d}s< + \infty $$

for each $i \in \{1,2,\ldots,m\}$, $j \in \{1,2,\ldots, n \}$. This assumption was used as a foundation to show the exponential stability in [2, 11, 12, 14–16]. In this paper, we explore a generalized Hanalay inequality by which we show that the solution of (1) is still asymptotically stable without this assumption.

The main results of this paper are the following two theorems.

### Theorem 1.1

*Suppose* $T_{1}$-$T_{5}$ *hold*, *then system* (1) *has a pap solution in the region*

$$B^{\ast }= \biggl\{ \varphi \in B \vert \Vert \varphi - \varphi_{0}\Vert _{B} \leq \frac{\delta L}{1-\delta } \biggr\} , $$

*where* $\varphi_{0}=\{\int_{-\infty }^{t}\mathrm{e}^{\int_{s}^{t}-a _{ij}(\eta)\,\mathrm{d}\eta }L_{ij}(s)\,\mathrm{d}s\}$, *and* *B* *is the pap* $m\times n$ *matrix valued function space*.

### Theorem 1.2

*Let all the conditions of Theorem* 1.1 *hold*. *Then the pap* $\varphi^{\ast }(t)=\{x_{ij}^{\ast }(t)\}$ *for system* (1) *in* $B^{\ast }$ *is globally asymptotically stable*, *i*.*e*. *for any other solution* $x(t)=\{x_{ij}(t)\}$ *for system* (1) *with initial conditions* $x_{ij}(t)=\varphi_{ij}(t)$, $t\in (-\infty,0]$, $\varphi_{ij}(t)$ *is continuous and bounded on* $(-\infty,0]$, $i\in \{1,2,\ldots,m\}$, $j\in \{1,2,\ldots,n\}$,

$$\lim_{t\rightarrow +\infty } \bigl\vert x(t)-\varphi^{\ast }(t) \bigr\vert =0. $$

This paper is organized as follows. The next section is on preliminaries including the definition and properties of pap functions, boundness of the output of (1) and the generalized Halanay inequality. The third section is devoted to the proof of our main results. An illustrative simulation example is given in the fourth section. We also give a conclusion in the last section.

## Preliminaries

Let $BC(\mathbb{R};\mathbb{R})$ denote the set of all bounded and continuous functions from $\mathbb{R}$ to itself. Throughout the rest of this paper, *i* and *j* are in the sets $\{1,2,\ldots,m\}$ and $\{1,2,\ldots,n\}$, respectively, unless otherwise stated.

### Definition 2.1

[20]

Denote by $\mathit{PAP}_{0}(\mathbb{R};\mathbb{R})$ the set of all such functions $\varphi \in BC(\mathbb{R};\mathbb{R})$, for which

$$\lim_{t\rightarrow \infty }\frac{1}{2t} \int_{-t}^{t} \bigl\vert \varphi (s) \bigr\vert \,\mathrm{d}s=0. $$

A function $f\in BC(\mathbb{R};\mathbb{R})$ is called pseudo almost periodic if $f=g+\varphi $, where $g\in AP(\mathbb{R};\mathbb{R})$, the space of almost periodic functions, and $\varphi \in \mathit{PAP}_{0}( \mathbb{R};\mathbb{R})$. Denote by $\mathit{PAP}(\mathbb{R};\mathbb{R})$ the set of all such functions.

### Definition 2.2

[20]

A function $f\in BC(\mathbb{R},\mathbb{R})$ is said to be ergodic if $\lim_{\alpha \rightarrow \infty }\frac{1}{2 \alpha }\int_{-\alpha }^{+\alpha }f(t+s)\,\mathrm{d}s=M(f)$ exists uniformly with respect to $t\in \mathbb{R}$.

We set $\{x_{ij}(t)\}=(x_{11}(t),\ldots,x_{1n}(t),\ldots,x_{i1}(t), \ldots,x_{in}(t),\ldots,x_{m1}(t),\ldots,x_{mn}(t))$. For each $x(t)=\{x_{ij}(t)\} \in \mathbb{R}^{m\times n}$, define the norm $\vert x(t)\vert =\max_{i,j}\{\vert x_{ij}(t)\vert \}$. Let $B= \{\varphi \vert \varphi (t) = \{\varphi_{ij}(t)\} \}$, where $\varphi_{ij}(t) \in \mathit{PAP}(\mathbb{R};\mathbb{R})$. For each $\varphi \in B $, if we define the induced module $\Vert \varphi \Vert _{B} = \sup_{t\in \mathbb{R}} \vert \varphi (t)\vert $, then *B* is a Banach space.

### Lemma 2.3

[20], Lemma 3.1.9

*Let* $a(t)\in \mathit{PAP}(\mathbb{R;\mathbb{R}})$ *be ergodic*. *If* $M(a)<0$ *and* $f(t)\in \mathit{PAP}(\mathbb{R};\mathbb{R})$ *then*

$$x'(t)=a(t)x(t)+f(t) $$

*has a unique bounded solution*

$$x(t)= \int_{-\infty }^{t}\mathrm{e}^{\int_{s}^{t}a(\eta)\,\mathrm{d} \eta }f(s) \, \mathrm{d}s, $$

*which is also in* $\mathit{PAP}(\mathbb{R};\mathbb{R})$.

### Lemma 2.4

[28],Proposition 5.3.2

*Suppose that* $k\in L^{1}( \mathbb{R};\mathbb{R})$ *and* $f\in \mathit{PAP}(\mathbb{R};\mathbb{R})$. *Then* $k\ast f\in \mathit{PAP}(\mathbb{R};\mathbb{R})$.

### Lemma 2.5

*Suppose that* $T_{1}$-$T_{5}$ *hold*. *Then solutions for initial value problem of system* (1) *are all bounded*.

### Proof

If $x_{ij}(t)>0$ for some *t*, we have

2 $$\begin{aligned} \frac{\mathrm{d}x_{ij}(t)}{\mathrm{d}t} \leq & -\underline{a}_{ij}x _{ij}(t)+M_{f}\hat{k}_{ij}\sum _{C_{kl}\in N_{r}(i,j)}\bar{C} _{ij}^{kl}x_{ij}(t)+L^{+}_{ij} \\ =& \biggl(-\underline{a}_{ij}+M_{f}\hat{k}_{ij} \sum_{C_{kl}\in N_{r}(i,j)}\bar{C}_{ij}^{kl} \biggr)x_{ij}(t)+L^{+} _{ij}. \end{aligned}$$

If $x_{ij}(t)<0$ for some *t*, we have

3 $$\begin{aligned} \frac{\mathrm{d}x_{ij}(t)}{\mathrm{d}t} \geq& -\underline{a}_{ij}x_{ij}(t)+M _{f}\hat{k}_{ij}\sum_{C_{kl}\in N_{r}(i,j)} \bar{C}_{ij}^{kl}x _{ij}(t)-L^{+}_{ij} \\ =& \biggl(-\underline{a}_{ij}+M_{f}\hat{k}_{ij} \sum_{C_{kl}\in N_{r}(i,j)}\bar{C}_{ij}^{kl} \biggr)x_{ij}(t)-L^{+} _{ij}. \end{aligned}$$

For $i\in \{1,2,\ldots,m\}$, $j\in \{1,2,\ldots,n\}$, since $\delta <1$,

4 $$\begin{aligned} -\underline{a}_{ij}+M_{f} \hat{k}_{ij}\sum_{C_{kl}\in N_{r}(i,j)} \bar{C}_{ij}^{kl}< 0. \end{aligned}$$

It follows from (2)-(4) and by the comparison principle that the solutions for initial value problems of system (1) are all bounded. □

### Lemma 2.6

Generalized Halanay inequality

*Let* $\eta >0$, $a_{i}>b_{i}>0$, $i=1,2,\ldots,n$. *Suppose* $x(t)$ *be a nonnegative continuous function on* $[t_{0}-\tau,t_{0}]$ *and satisfy the inequality*:

5 $$\begin{aligned} D^{+}x(t)\leq -a_{i}x(t)+b_{i} \bar{x}(t)+\eta \end{aligned}$$

*for* $t\ge t_{0}$, *where* $D^{+}$ *is the upper right derivative*, $\bar{x}(t)=\sup_{t-\tau \leq s\leq t}x(s)$, *τ* *is a positive constant*, *the subscript* *i* *of* $a_{i}$ *and* $b_{i}$ *varies piecewise with* $t\in \mathbb{R}$, *then the following inequality holds*:

6 $$\begin{aligned} x(t)\leq \max_{1\leq i \leq n} \biggl\{ \sup _{t_{0}-\tau \leq s \leq t_{0}} \biggl\vert x(s)-\frac{\eta }{a_{i}-b_{i}} \biggr\vert \biggr\} \cdot \mathrm{e}^{-\lambda_{\min }(t-t _{0})} +\max_{1\leq i \leq n} \biggl\{ \frac{\eta }{a_{i}-b_{i}} \biggr\} , \end{aligned}$$

*where* $t \ge t_{0}$, $\lambda_{\min }=\min_{1\leq i \leq n} \{ \lambda_{i} \} $ *and* $\lambda_{i}$ *is the unique positive solution of the equation*:

7 $$\begin{aligned} \lambda_{i}=a_{i}-b_{i} \mathrm{e}^{\lambda_{i} \tau }. \end{aligned}$$

### Proof

First, we prove that equation (7) has a unique positive solution. Consider the function:

$$f(\mu)=\mu -a_{i}+b_{i}\mathrm{e}^{\mu \tau }, \quad \mu \in [0,a_{i}]. $$

Since $f(0)=-a_{i}+b_{i}<0$, $f(a_{i})=b_{i}\mathrm{e}^{a_{i}\tau }>0$, $f'(\mu)=1+b_{i}\tau \mathrm{e}^{\mu \tau }>0$, there is a unique $\lambda_{i} \in [0,a_{i}]$ which satisfies equation (7).

Denote $Z(t)=\max_{1\leq i \leq n} \{ \sup_{t_{0}-\tau \leq s \leq t _{0}}\vert x(s)-\frac{\eta }{a_{i}-b_{i}}\vert \} \cdot \mathrm{e}^{- \lambda_{\min }(t-t_{0})}$. Obviously, inequality (6) holds when $t_{0}-\tau \leq t \leq t_{0}$. Let $C>1$, now we will show that

8 $$\begin{aligned} x(t)\leq CZ(t)+\max_{1\leq i \leq n} \biggl\{ \frac{\eta }{a_{i}-b _{i}} \biggr\} , \end{aligned}$$

when $t\geq t_{0}$.

Otherwise, there exist $t_{1}$, $\beta \in \mathbb{R}$ such that $t_{0}< t_{1}<\beta $,

9 $$\begin{aligned} x(t)\leq CZ(t)+\max_{1 \leq i \leq n} \biggl\{ \frac{\eta }{a_{i}-b _{i}} \biggr\} ,\quad \mbox{when } t_{0}-\tau \leq t< t_{1} \end{aligned}$$

and

10 $$\begin{aligned}& \begin{aligned}&x(t_{1})= CZ(t_{1})+\max _{1 \leq i \leq n} \biggl\{ \frac{\eta }{a _{i}-b_{i}} \biggr\} ; \\ & x(t)>CZ(t)+ \max_{1 \leq i \leq n} \biggl\{ \frac{ \eta }{a_{i}-b_{i}} \biggr\} \end{aligned} \end{aligned}$$

when $t_{1}< t<\beta $. It follows from (5), (9) and (10) that

11 $$\begin{aligned} D^{+}x(t_{1}) \leq & -a_{i}x(t_{1})+b_{i} \bar{x}(t_{1})+\eta \\ \leq & -a_{i} \biggl( CZ(t_{1})+\max_{1 \leq i \leq n} \biggl\{ \frac{ \eta }{a_{i}-b_{i}} \biggr\} \biggr) +b_{i} \biggl( CZ(t_{1}-\tau)+\max_{1 \leq i \leq n} \biggl\{ \frac{\eta }{a_{i}-b_{i}} \biggr\} \biggr) + \eta \\ \leq & C \bigl(-a_{i}+b_{i}\mathrm{e}^{\lambda_{\min }\tau } \bigr)Z(t_{1})- \biggl( (a_{i}-b_{i})\max _{1 \leq i \leq n} \biggl\{ \frac{1}{a_{i}-b _{i}} \biggr\} -1 \biggr) \eta \\ \leq & C \bigl(-a_{i}+b_{i}\mathrm{e}^{\lambda_{i}\tau } \bigr)Z(t_{1}) \\ =&C(- \lambda_{i})Z(t_{1}) \\ \leq & C(-\lambda_{\min })Z(t_{1})=CZ'(t_{1}). \end{aligned}$$

Obviously (11) contradicts (10), and this implies that (8) holds on $\mathbb{R}$. Since $C>1$ is arbitrary, (6) holds. This completes the proof. □

## Proof of main results

In this section, we give the proof of our main results Theorem 1.1 and 1.2 in detail.

### The proof of Theorem 1.1

Obviously $B^{\ast }$ is a closed subset of *B*. For $\forall \varphi \in B$, consider the solution $x_{\varphi }(t)$ of differential equation

$$\begin{aligned} \frac{\mathrm{d}x_{ij}}{\mathrm{d}t} =&-a_{ij}(t)x_{ij}- \sum_{C_{kl}\in N_{r}(i,j)}C_{ij}^{kl}(t) \int_{0}^{+\infty }k_{ij}(u)f \bigl( \varphi_{kl}(t-u) \bigr)\,\mathrm{d}u\, \varphi_{ij}(t) +L_{ij}(t). \end{aligned}$$

Since *f* is Lipschitz continuous and $\varphi_{kl}(t)\in \mathit{PAP}( \mathbb{R};\mathbb{R})$, $f(\varphi_{kl}(t))\in \mathit{PAP}(\mathbb{R}; \mathbb{R})$ (see Corollary 1.5.4 in [20]). Lemma 2.4 implies $\int_{0}^{+\infty }k_{ij}(u)f( \varphi_{kl}(t-u))\,\mathrm{d}u\in \mathit{PAP}(\mathbb{R};\mathbb{R})$. It follows from Lemma 2.3 that (12) has a unique pseudo almost periodic solution

$$\begin{aligned} x_{\varphi }(t) =& \biggl\{ \int_{-\infty }^{t}\mathrm{e}^{\int_{s}^{t}-a _{ij}(\eta)\,\mathrm{d}\eta } \biggl[-\sum _{C_{kl}\in N_{r}(i,j)}C _{ij}^{kl}(s) \int_{0}^{+\infty }k_{ij}(u)f \bigl( \varphi_{kl}(s-u) \bigr) \,\mathrm{d}u\, \varphi_{ij}(s) +L_{ij}(s) \biggr]\,\mathrm{d}s \biggr\} . \end{aligned}$$

Define the mapping $T:B\rightarrow B$ by $(T\varphi)(t)=x_{\varphi }(t)$.

$$\begin{aligned} \Vert \varphi_{0}\Vert _{B}=\sup _{t\in \mathbb{R}}\max_{(i,j)} \biggl\{ \biggl\vert \int_{-\infty }^{t}\mathrm{e}^{\int_{s}^{t}-a_{ij}( \eta)\,\mathrm{d}\eta }L_{ij}(s) \,\mathrm{d}s \biggr\vert \biggr\} \leq \sup_{t\in \mathbb{R}}\max _{(i,j)} \biggl\{ \frac{L^{+} _{ij}}{\underline{a}_{ij}} \biggr\} =\max _{(i,j)} \biggl\{ \frac{L^{+}_{ij}}{\underline{a}_{ij}} \biggr\} =L. \end{aligned}$$

Thus, for all $\varphi \in B^{\ast }$, we have

12 $$\begin{aligned} \Vert \varphi \Vert _{B}\leq \Vert \varphi -\varphi_{0}\Vert _{B}+\Vert \varphi_{0}\Vert _{B} \leq \frac{L}{1-\delta }. \end{aligned}$$

Now, we prove that the mapping *T* is a self-mapping of $B^{\ast }$. In fact, for all $\varphi \in B^{\ast }$, together with (12), we obtain

$$\begin{aligned}& \Vert T\varphi -\varphi_{0}\Vert _{B} \\& \quad =\sup_{t\in \mathbb{R}}\max_{(i,j)} \biggl\{ \biggl\vert \int_{-\infty }^{t}\mathrm{e}^{\int_{s}^{t}-a_{ij}(\eta)\,\mathrm{d} \eta }\sum _{C_{kl}\in N_{r}(i,j)}C_{ij}^{kl}(t) \int_{0}^{+ \infty }k_{ij}(u)f \bigl( \varphi_{kl}(s-u) \bigr)\,\mathrm{d}u\, \varphi_{ij}(s) \, \mathrm{d}s \biggr\vert \biggr\} \\& \quad \leq \sup_{t\in \mathbb{R}}\max_{(i,j)} \biggl\{ \int_{-\infty }^{t}\mathrm{e}^{\int_{s}^{t}-a_{ij}(\eta)\,\mathrm{d} \eta }\sum _{C_{kl}\in N_{r}(i,j)}C_{ij}^{kl}(s) \int_{0}^{+ \infty } \bigl\vert k_{ij}(u) \bigr\vert M_{f}\,\mathrm{d}u \bigl\vert \varphi (s) \bigr\vert \, \mathrm{d}s \biggr\} \\& \quad \leq \sup_{t\in \mathbb{R}}\max_{(i,j)} \biggl\{ \int_{-\infty }^{t}\mathrm{e}^{\int_{s}^{t}-a_{ij}(\eta)\,\mathrm{d} \eta }M_{f} \hat{k}_{ij}\sum_{C_{kl}\in N_{r}(i,j)}\bar{C}_{ij} ^{kl}\frac{L}{1-\delta }\,\mathrm{d}s \biggr\} \\& \quad \leq \max_{(i,j)} \biggl\{ \frac{M_{f}\hat{k}_{ij}\sum_{C_{kl}\in N_{r}(i,j)}\bar{C}_{ij}^{kl}}{\underline{a}_{ij}} \biggr\} \frac{L}{1-\delta } =\frac{\delta L}{1-\delta } . \end{aligned}$$

This shows that $(T\varphi)\in B^{\ast }$. Next, we prove that the mapping *T* is a contraction on $B^{\ast }$. In fact, for all $\varphi, \psi \in B^{\ast }$, we have

$$\begin{aligned}& \Vert T\varphi -T\psi \Vert _{B} \\& \quad =\sup_{t\in \mathbb{R}} \bigl\vert (T\varphi) (t)-(T\psi) (t) \bigr\vert \\& \quad =\sup_{t \in \mathbb{R}}\max_{(i,j)} \biggl\{ \biggl\vert \int_{-\infty }^{t}\mathrm{e}^{\int_{s}^{t}-a_{ij}(\eta)\,\mathrm{d} \eta }\sum _{C_{kl}\in N_{r}(i,j)}C_{ij}^{kl}(s) \biggl[ \int_{0} ^{+\infty }k_{ij}(u)f \bigl( \varphi_{kl}(s-u) \bigr)\,\mathrm{d}u \,\varphi_{ij}(s) \\& \qquad {}- \int_{0}^{+\infty }k_{ij}(u)f \bigl( \psi_{kl}(s-u) \bigr)\,\mathrm{d}u\, \psi_{ij}(s) \biggr]\, \mathrm{d}s \biggr\vert \biggr\} \\& \quad =\sup_{t \in \mathbb{R}}\max_{(i,j)} \biggl\{ \biggl\vert \int_{-\infty }^{t}\mathrm{e}^{\int_{s}^{t}-a_{ij}(\eta)\,\mathrm{d} \eta }\sum _{C_{kl}\in N_{r}(i,j)}C_{ij}^{kl}(s) \biggl[ \int_{0} ^{+\infty }k_{ij}(u) \bigl(f \bigl( \varphi_{kl}(s-u) \bigr) \\& \qquad {} -f \bigl(\psi_{kl}(t-u) \bigr) \bigr)\,\mathrm{d}u\, \varphi_{ij}(s)+ \int_{0}^{+\infty }k _{ij}(u)f \bigl( \psi_{kl}(s-u) \bigr)\,\mathrm{d}u \bigl(\varphi_{ij}(s)- \psi_{ij}(s) \bigr) \biggr]\,\mathrm{d}s \biggr\vert \biggr\} . \end{aligned}$$

In view of (12) and the equality above, we have

$$\begin{aligned}& \Vert T\varphi -T\psi \Vert _{B} \\& \quad \leq \sup_{t \in \mathbb{R}}\max_{(i,j)} \biggl\{ \biggl\vert \int_{-\infty }^{t}\mathrm{e}^{\int_{s}^{t}-a_{ij} (\eta) \,\mathrm{d}\eta }\sum _{C_{kl}\in N_{r}(i,j)}\bar{C}_{ij}^{kl} \biggl[ \int _{0}^{+\infty }\bigl\vert k_{ij}(u)\bigr\vert \mu \bigl\vert \varphi_{kl}(s-u) \\& \qquad {} -\psi_{kl}(s-u)\bigr\vert \mathrm{d}u \bigl\vert \varphi (s)\bigr\vert + \int_{0}^{+\infty } \bigl\vert k_{ij}(u) \bigr\vert M _{f}\,\mathrm{d}u \bigl\vert \varphi_{ij}(s)- \psi_{ij}(s) \bigr\vert \biggr]\,\mathrm{d}s \biggr\vert \biggr\} \\& \quad \leq \sup_{t \in \mathbb{R}}\max_{(i,j)} \biggl\{ \int_{-\infty }^{t}\mathrm{e}^{\int_{s}^{t}-a_{ij}(\eta)\,\mathrm{d} \eta }\sum _{C_{kl}\in N_{r}(i,j)}\bar{C}_{ij}^{kl} \biggl(\hat{k} _{ij}\frac{\mu L}{1-\delta }+\hat{k}_{ij}M_{f} \biggr) \,\mathrm{d}s \biggr\} \Vert \varphi -\psi \Vert _{B} \\& \quad \leq \max_{(i,j)} \biggl\{ \frac{M_{f}\hat{k}_{ij}\sum_{C_{kl}\in N_{r}(i,j)}\bar{C}_{ij}^{kl}(1+\frac{\mu L}{(1- \delta)M_{f}})}{\underline{a}_{ij}} \biggr\} \Vert \varphi -\psi \Vert _{B} =q\Vert \varphi -\psi \Vert _{B}. \end{aligned}$$

Note that $q<1$, the mapping *T* is a contraction. Therefore, the mapping *T* possesses a unique fixed point $\varphi^{\ast }\in B^{ \ast }$, $T\varphi^{\ast }=\varphi^{\ast }$. By (12), $\varphi^{\ast }$ satisfies (1), so $\varphi^{\ast }$ is a pseudo almost periodic solution of system (1) in $B^{\ast }$. The proof is complete. □

### The proof of Theorem 1.2

Since $q<1$ in $T_{5}$, we can get easily

13 $$\begin{aligned} \underline{a}_{ij}-M_{f} \hat{k}_{ij}\sum_{C_{kl}\in N_{r}(i,j)} \bar{C}_{ij}^{kl}> \frac{\mu L}{1-\delta } \hat{k}_{ij}\sum_{C_{kl}\in N_{r}(i,j)} \bar{C}_{ij}^{kl}>0. \end{aligned}$$

By Lemma 2.5, $x(t)=\{x_{ij}(t)\}$ is bounded on $\mathbb{R}$. We denote $M_{\varphi }=\sup_{t\in \mathbb{R}}\vert x(t)\vert $.

Let $y(t)=\{y_{ij}(t)\}=\{x_{ij}(t)-x^{\ast }_{ij}(t)\}=x(t)- \varphi^{\ast }(t)$, then

14 $$\begin{aligned} \frac{\mathrm{d}y_{ij}(t)}{\mathrm{d}t} =&-a_{ij}(t)y_{ij}(t)- \biggl( \sum_{C_{kl}\in N_{r}(i,j)}C_{ij}^{kl}(t) \int_{0}^{+\infty }k _{ij}(u)f \bigl(x_{kl}(t-u) \bigr)\,\mathrm{d}u\, x_{ij}(t) \\ &{}-\sum_{C_{kl}\in N_{r}(i,j)}C_{ij}^{kl}(t) \int_{0}^{+\infty }k_{ij}(u)f \bigl(x^{\ast }_{kl}(t-u) \bigr)\,\mathrm{d}u\, x^{\ast }_{ij}(t) \biggr). \end{aligned}$$

For convenience, denote $\vert y(t)\vert =\vert y_{i_{0}j_{0}}(t)\vert $. Since $y(t)$ is continuous, $i_{0}$ and $j_{0}$ vary piecewise with $t\in \mathbb{R}$. In view of (14), we have

$$\begin{aligned}& D^{+} \bigl\vert y(t) \bigr\vert =D^{+} \bigl\vert y_{i_{0}j_{0}}(t) \bigr\vert \\& \quad =-a_{i_{0}j_{0}}(t) \bigl\vert y_{i_{0}j_{0}}(t) \bigr\vert \\& \qquad {} - \operatorname{sgn}\bigl(y_{i_{0}j_{0}}(t) \bigr) \biggl\{ \sum _{C_{kl}\in N_{r}(i_{0},j_{0})}C_{i_{0}j_{0}}^{kl}(t) \int_{0}^{+\infty }k_{i_{0}j_{0}}(u) f \bigl(x_{kl}(t-u) \bigr)\,\mathrm{d}u x_{i _{0}j_{0}}(t) \\& \qquad {}-\sum_{C_{kl}\in N_{r}(i_{0},j_{0})}C_{i_{0}j_{0}}^{kl}(t) \int_{0}^{+\infty } k_{i_{0}j_{0}}(u)f \bigl(x^{\ast }_{kl}(t-u) \bigr)\,\mathrm{d}u \,x^{\ast }_{i_{0}j_{0}}(t) \biggr\} \\& \quad =-a_{i_{0}j_{0}}(t) \bigl\vert y_{i_{0}j_{0}}(t) \bigr\vert \\& \qquad {} - \operatorname{sgn}\bigl(y_{i_{0}j_{0}}(t) \bigr)\biggl\{ \sum_{C_{kl}\in N_{r}(i_{0},j_{0})}C_{i_{0}j_{0}} ^{kl}(t) \int_{0}^{+\infty }k_{i_{0}j_{0}}(u)f \bigl(x_{kl}(t-u) \bigr)\,\mathrm{d}u \bigl(x_{i_{0}j_{0}}(t)-x^{\ast }_{i_{0}j_{0}}(t) \bigr) \\& \qquad {}+\sum_{C_{kl}\in N_{r}(i_{0},j_{0})}C_{i_{0}j_{0}}^{kl}(t) \int_{0}^{+\infty }k_{i_{0}j_{0}}(u) \bigl(f \bigl(x_{kl}(t-u) \bigr)-f \bigl(x^{\ast }_{kl}(t-u) \bigr) \bigr) \,\mathrm{d}u \,x^{\ast }_{i_{0}j_{0}}(t) \biggr\} \\& \quad \leq -\underline{a}_{i_{0}j_{0}} \bigl\vert y(t) \bigr\vert +\sum _{C_{kl}\in N_{r}(i_{0},j_{0})}\bar{C}_{i_{0}j_{0}}^{kl} \int _{0}^{+\infty } \bigl\vert k_{i_{0}j_{0}}(u) \bigr\vert \bigl\vert f \bigl(x_{kl}(t-u) \bigr) \bigr\vert \, \mathrm{d}u \bigl\vert y_{i_{0}j_{0}}(t) \bigr\vert \\& \qquad {}+\sum_{C_{kl}\in N_{r}(i_{0},j_{0})}\bar{C}_{i_{0}j_{0}}^{kl} \int_{0}^{+\infty } \bigl\vert k_{i_{0}j_{0}}(u) \bigr\vert \mu \bigl\vert y_{kl}(t-u) \bigr\vert \bigl\vert x^{\ast }_{i_{0}j_{0}}(t) \bigr\vert \,\mathrm{d}u \\& \quad \leq -\underline{a}_{i_{0}j_{0}} \bigl\vert y(t) \bigr\vert +\sum _{C_{kl}\in N_{r}(i_{0},j_{0})}\bar{C}_{i_{0}j_{0}}^{kl} M_{f} \hat{k}_{i_{0}j_{0}} \bigl\vert y(t) \bigr\vert \\& \qquad {}+\sum _{C_{kl}\in N_{r}(i_{0},j_{0})} \bar{C}_{i_{0}j_{0}}^{kl} \frac{\mu L}{1-\delta } \int_{0}^{+\infty } \bigl\vert k_{i_{0}j_{0}}(u) \bigr\vert \bigl\vert y(t-u) \bigr\vert \,\mathrm{d}u \\& \quad = \biggl(-\underline{a}_{ij}+M_{f}\hat{k}_{i_{0}j_{0}} \sum_{C_{kl}\in N_{r}(i_{0},j_{0})}\bar{C}_{i_{0}j_{0}}^{kl} \biggr) \bigl\vert y(t) \bigr\vert \\& \qquad {} +\frac{\mu L}{1-\delta }\sum _{C_{kl}\in N_{r}(i_{0},j_{0})} \bar{C}_{i_{0}j_{0}}^{kl} \int_{0}^{+\infty } \bigl\vert k_{i_{0}j_{0}}(u) \bigr\vert \bigl\vert y(t-u) \bigr\vert \,\mathrm{d}u. \end{aligned}$$

For any $\epsilon >0$, there exists a positive number $T>0$ such that

$$\begin{aligned}& \frac{\mu L}{1-\delta }\sum_{C_{kl}\in N_{r}(i,j)}\bar{C} _{ij}^{kl} \int_{T}^{+\infty } \bigl\vert k_{ij}(u) \bigr\vert \bigl\vert y(t-u) \bigr\vert \,\mathrm{d}u \\& \quad \leq \frac{\mu L}{1-\delta }\sum_{C_{kl}\in N_{r}(i,j)} \bar{C}_{ij}^{kl} \int_{T}^{+\infty } \bigl\vert k_{ij}(u) \bigr\vert \biggl(M_{\varphi } +\frac{L}{1- \delta } \biggr)\,\mathrm{d}u \leq \epsilon. \end{aligned}$$

From the last two inequality systems, we get

15 $$\begin{aligned} D^{+} \bigl\vert y(t) \bigr\vert \leq & \biggl(- \underline{a}_{i_{0}j_{0}}+M_{f}\hat{k}_{i _{0}j_{0}}\sum _{C_{kl}\in N_{r}(i_{0},j_{0})} \bar{C}_{i_{0}j _{0}}^{kl} \biggr) \bigl\vert y(t) \bigr\vert \\ &{}+\frac{\mu L}{1-\delta }\sum_{C_{kl}\in N_{r}(i_{0},j_{0})} \bar{C}_{i_{0}j_{0}}^{kl} \int_{0}^{T} \bigl\vert k_{i_{0}j_{0}}(u) \bigr\vert \bigl\vert y(t-u) \bigr\vert \,\mathrm{d}u \\ &{}+\frac{\mu L}{1-\delta }\sum_{C_{kl}\in N_{r}(i_{0},j_{0})} \bar{C}_{i_{0}j_{0}}^{kl} \int_{T}^{+\infty } \bigl\vert k_{i_{0}j_{0}}(u) \bigr\vert \bigl\vert y(t-u) \bigr\vert \,\mathrm{d}u \\ \leq & \biggl(-\underline{a}_{i_{0}j_{0}}+M_{f} \hat{k}_{i_{0}j_{0}} \sum_{C_{kl}\in N_{r}(i_{0},j_{0})} \bar{C}_{i_{0}j_{0}}^{kl} \biggr) \bigl\vert y(t) \bigr\vert \\ &{}+\frac{\mu L}{1-\delta }\hat{k}_{i_{0}j_{0}}\sum_{C_{kl}\in N_{r}(i_{0},j_{0})} \bar{C}_{i_{0}j_{0}}^{kl} \max_{t-T\leq s\leq t} \bigl\vert y(s) \bigr\vert +\epsilon. \end{aligned}$$

By Lemma 2.6, it follows from (15) that

16 $$\begin{aligned} \bigl\vert y(t) \bigr\vert \leq & \max_{(i_{0},j_{0})} \biggl\{ \sup_{-T\leq s\leq 0} \biggl\vert y(s)-\frac{\epsilon }{A_{i_{0}j_{0}}-B _{i_{0}j_{0}}} \biggr\vert \biggr\} \cdot \mathrm{e}^{-\lambda t} + \max_{(i_{0},j_{0})} \biggl\{ \frac{\epsilon }{A_{i_{0}j_{0}}-B_{i _{0}j_{0}}} \biggr\} \\ \leq & \max_{(i_{0},j_{0})} \biggl\{ \sup_{-\infty \leq s\leq 0} \biggl\vert y(s)-\frac{\epsilon }{A_{i_{0}j _{0}}-B_{i_{0}j_{0}}} \biggr\vert \biggr\} \cdot \mathrm{e}^{-\lambda t} + \max_{(i_{0},j_{0})} \biggl\{ \frac{\epsilon }{A_{i_{0}j_{0}}-B _{i_{0}j_{0}}} \biggr\} \\ \leq & \max_{(i_{0},j_{0})} \biggl\{ M_{\varphi }+ \frac{L}{1- \delta }+\frac{\epsilon }{A_{i_{0}j_{0}}-B_{i_{0}j_{0}}} \biggr\} \cdot \mathrm{e}^{-\lambda t}+ \max_{(i_{0},j_{0})} \biggl\{ \frac{ \epsilon }{A_{i_{0}j_{0}}-B_{i_{0}j_{0}}} \biggr\} , \end{aligned}$$

where $t\geq -T$, $A_{i_{0}j_{0}}=\underline{a}_{i_{0}j_{0}}-M_{f} \hat{k}_{i_{0}j_{0}}\sum_{C_{kl}\in N_{r}(i_{0},j_{0})}\bar{C} _{i_{0}j_{0}}^{kl}$, $B_{i_{0}j_{0}}=\frac{\mu L}{1-\delta }\hat{k} _{i_{0}j_{0}}\sum_{C_{kl}\in N_{r}(i_{0},j_{0})}\bar{C}_{i_{0}j _{0}}^{kl}$ and $\lambda =\min_{(i_{0},j_{0})} \{ \lambda_{i_{0}j _{0}} \} $ and $\lambda_{i_{0}j_{0}}$ is the unique positive number which satisfies the following equation:

$$\lambda_{i_{0}j_{0}}=A_{i_{0}j_{0}}-B_{i_{0}j_{0}}\mathrm{e}^{ \lambda_{i_{0}j_{0}} T}. $$

Because *ϵ* is arbitrary, we get from (16) that $\vert y(t)\vert \rightarrow 0$, $t\rightarrow +\infty$. The proof is complete. □

### Remark 3.1

As we pointed out early that in order to show the exponential stability of the solutions for system (1), many authors use the assumption $T_{0}$. But we still can get the globally asymptotical stability of the solutions in Theorem 1.2 without $T_{0}$. More precisely, we can get the conclusion from (16) that, for any given $\varepsilon >0$, solutions of initial value problem of (1) will converge exponentially to $\varphi^{\ast }$ with error $\mathcal{O}(\varepsilon)$ where the exponent is determined by *ε*.

## An example

In this section, we construct an example to demonstrate the results obtained in the previous sections.

### Example 4.1

We consider the SICNNs model (1) with $3\times 3$ lattice. Set $r=1$, the activity function $f(x)=\frac{1}{20}(\vert x-1\vert -\vert x+1\vert )$, and the delay kernel $k_{ij}(u)=\frac{1}{(\ln 2) (t+2)[\ln (t+2)]^{2}}$.

We set the decay rate

$$\bigl[a_{ij}(t) \bigr]_{3\times 3}= \left[ \begin{matrix} 3+\sin t+\exp {(-\vert t\vert )} & 3+\cos t+\exp {(-\vert t\vert )} & 4+\sin \sqrt{2}t \\ 3+\exp {(-t^{2})} & 2+\cos \sqrt{2}t & 3+\sin t+ \exp {(-\vert t\vert )} \\ 4-\sin t & 1+\exp {(-t^{2})} & 4+\cos t \end{matrix}\right] , $$

the connection strength matrix

$$\bigl[c_{ij}(t) \bigr]_{3\times 3}= \left[ \begin{matrix} 0.1\vert \sin t\vert & 0.2\vert \sin \sqrt{2}t\vert & 0.1 \sin^{2} t \\ 0.2\vert \cos t\vert & 0 & 0.2\cos^{2} t \\ 0.1\cos^{2}\sqrt{3}t & 0.2\vert \sin \sqrt{3}t\vert & 0.1\vert \cos t\vert \end{matrix}\right] , $$

the input signals

$$\bigl[L_{ij}(t) \bigr]_{3\times 3}= \left[ \begin{matrix} 0.7+0.2 \sin^{2} t + 0.1\frac{1}{t^{2}+1} & 0.4+0.3\cos^{2}{t} & 0.9+ \sin {t} \\ 0.9+2\cos^{2}{t} & 0.6+0.2\sin^{2}{t} & 0.8+\sin^{2}{t} \\ 0.4+2\cos^{4}{t} & 0.5+0.4\sin^{2}{t}+\exp {-t^{2}} & 0.9+\frac{2}{t ^{4}+1} \end{matrix}\right] . $$

It is clearly that the coefficients and input signals are all pap and $a_{ij}(\cdot)$ is ergodic. By direct calculation, we obtain $\hat{k}_{ij}=1$, $M_{f}=1$, $\mu =0.1$, and

$$\begin{aligned}& [\underline{a}_{ij}]_{3\times 3}= \left[ \begin{matrix}2 & 2 & 3 \\ 3 & 1 & 2 \\ 3 & 1 & 3 \end{matrix}\right] , \\& \bigl[L_{ij}^{+} \bigr]_{3\times 3}= \left[ \begin{matrix} 1 & 0.7 & 1.9 \\ 2.9 & 0.8 & 1.8 \\ 2.4 & 1.9 & 2.9 \end{matrix}\right] , \\& \biggl[ \sum _{C_{kl}\in N_{1}(i,j)}\overline{C}_{ij}^{kl} \biggr] _{3\times 3} = \left[ \begin{matrix} 0.5 & 0.8 & 0.5 \\ 0.8 & 1.2 & 0.8 \\ 0.5 & 0.8 & 0.5 \end{matrix}\right] . \end{aligned}$$

The assumption $T_{5}$ is valid, more specifically,

$$\delta = \biggl\{ \frac{M_{f} \hat{k}_{22} \sum_{C_{kl}\in N_{1}(2,2)\overline{C}_{22}^{kl}}}{\underline{a}_{22}} \biggr\} =0.12< 1, \qquad L=\frac{L_{32}^{+}}{\underline{a}_{32}}=1.9, \quad \mbox{and}\quad q=0.146< 1. $$

**Figure 1:**
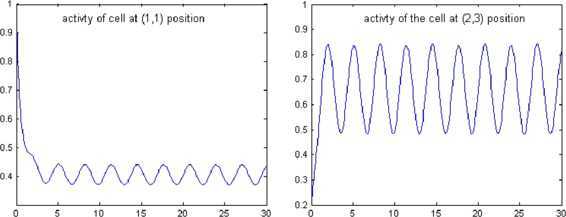
**Numerical solutions of** $\pmb{x_{11}(t)}$ **and** $\pmb{x_{23}(t)}$ **of Example** **4.1** **.**

Now Theorems 1.1 and 1.2 imply that this example SICNNs has a globally asymptotically stable pap solution. The fact is verified by the numerical simulation in Figure 1 where the initial value is

$$x(t)\equiv \left[ \begin{matrix}1 & 1 & 1 \\ 1 & 1 & 0.2 \\ 0.2 & 1 & 1 \end{matrix}\right] $$

for $t\leq 0$.

## Conclusion

In this paper, SICNNs with continuously distributed delays and pap coefficients and input signals have been studied. We have obtained some sufficient conditions for the existence and globally asymptotical stability of pseudo almost periodic solutions. These obtained results are new and complement previously known results since the decay rate and delay kernels considered here are more general. Moreover, an example is given to illustrate the effectiveness of our results.

The SICNNS with continuously distributed delays is a kind of integro-differential systems. Lyapunov functionals and differential inequality techniques are the main methods to deal with periodic and almost periodic solutions of SICNNs in the present literature. In [29, 30], a special method reducing system of n integro-differential equations to system of *m* ($m > n$) ordinary differential equations was presented. This idea opens new possibilities in the study of almost periodic and pap solutions to SICNNs with distributed delays. This should be one of our future research directions after this paper.
